# Hyperglycemia and Chemoresistance in Breast Cancer: From Cellular Mechanisms to Treatment Response

**DOI:** 10.3389/fonc.2021.628359

**Published:** 2021-02-25

**Authors:** Jie Qiu, Qinghui Zheng, Xuli Meng

**Affiliations:** ^1^ Zhejiang Chinese Medical University, Hangzhou, China; ^2^ Department of Breast Surgery, Zhejiang Provincial People’s Hospital, Hangzhou, China

**Keywords:** hyperglycemia, chemotherapy resistance, chemoresistance, breast cancer, glucose metabolism

## Abstract

Female breast cancer is a complex, multifactorial disease. Studies have shown that hyperglycemia is one of the most important contributing factors to increasing the risk of breast cancer that also has a major impact on the efficacy of chemotherapy. At the cellular level, hyperglycemia can promote the proliferation, invasion, and migration of breast cancer cells and can also induce anti-apoptotic responses to enhance the chemoresistance of tumors *via* abnormal glucose metabolism. In this article, we focus on the latest progress in defining the mechanisms of chemotherapy resistance in hyperglycemic patients including the abnormal behaviors of cancer cells in the hyperglycemic microenvironment and the impact of abnormal glucose metabolism on key signaling pathways. To better understand the advantages and challenges of breast cancer treatments, we explore the causes of drug resistance in hyperglycemic patients that may help to better inform the development of effective treatments.

## Background

Breast cancer (BC) is the most common malignancy in women all over the world (1) and has several known risk factors including age, sex, obesity, estrogen levels, and family history (2). Recent studies have shown that hyperglycemia is an important risk factor in the development of BC (3). In BC, hyperglycemia is associated with an increased prevalence and mortality but also has major impacts on the efficacy of chemotherapy efficacy and can lead to chemoresistance. The development and progression of BC involves the dysfunction of several molecular processes including abnormal glucose metabolism, abnormal insulin levels, insulin resistance, distorted signal pathways, oxidative stress, and enhanced inflammatory processes (4, 5).

The “Warburg” effect is considered to be the most important feature of glucose metabolism in tumors (6). Under hypoxic conditions, aerobic glycolysis in tumor cells significantly changes compared to aerobic oxidation and so cancer cells upregulate the processes of glycolysis and the catabolism of glucose to form lactate. This process is accompanied by ATP production. However, ATP produced by glycolysis is not sufficient to support the survival of cancer cells and so the rate of glucose uptake and the fermentation of glucose to lactate are both increased (7). Sufficient energy supply activates cellular signaling pathways, promotes the abnormal activity of tumor cells, and induces an anti-apoptotic response and chemotherapy resistance (8). The reprogramming of glucose metabolism accelerates the conversion of glycolysis and changes the acidity of the microenvironment which acts to promote the expression of angiogenic factors and enhance tumor metastasis (9).

Hyperglycemia in patients is mostly accompanied by dyslipidemia. It has been reported that glycolipid metabolism may have a synergistic influence on chemotherapy resistance (10). Obesity leads to an accumulation of lipids and increases the circulating levels of fatty acids that enhance insulin resistance and hyperinsulinemia eventually leading to hyperglycemia and diabetes (1). However, glucose also can act as a substrate for fatty acid synthase which is a key enzyme responsible for the *de novo* synthesis of fatty acids (11, 12). The prognosis for women with BC is adversely affected by the comorbidities of hyperlipidemia and hyperglycemia. In this review, we primarily focus on the effects of crucial glucose metabolic pathways to explore how abnormal blood glucose levels influence the pathology of BC cells and reduce the efficacy of chemotherapy to drive chemoresistance.

## The Effects of Hyperglycemia on Tumor Behavior

### Promotion of Proliferation and Metastasis

Hyperglycemia can provide nutrients for the rapid proliferation of breast cancer cells. Studies have reported that high concentrations of glucose significantly increase the proliferation of BC cells (such as MDA-MB-231 and MCF-7) (13, 14) through a mechanism involving activation of epidermal growth factor receptors (EGFRs) (15). EGFR is frequently overexpressed in triple-negative BC and is associated with poor prognosis. It has been reported that the GTPase activating protein (GAP) USP6NL that is involved in endocytosis and signal transduction is also overexpressed in BC, particularly the basal-like subtype (16). BC cells with high levels of USP6NL show delayed inactivation of EGFR leading to chronic activation of AKT which maintains the stability of the glucose transporter 1 (GLUT1) on the plasma membrane leading to increased glucose metabolism (17). GLUT1 transports and absorbs glucose through the plasma membrane to provide energy for BC cells and to promote cellular proliferation and invasion (18, 19). When BC cells lack sensitivity to respond to changes in glucose concentration, elevated USP6NL can compensate for this deficiency and stabilize GLUT1 by activated AKT, shows that its glycolysis ability depends on the protein.

Long-term hyperglycemia results in insulin resistance, hyperinsulinemia, and dysfunctional signaling through the insulin-like growth factor-1 pathway (20). It has been reported that IGF-1 participates in estrogen receptor signal transduction through IGF-1 receptor/ER interactions. This process is bidirectional as these interactions can regulate the proliferation, apoptosis, and differentiation of breast epithelial cells, resulting in increased risk of endocrine-related cancers particularly in post-menopausal women (21).

It has also been reported that hyperglycemia promotes the proliferation of malignant BC epithelial cells by increasing activity of the leptin/insulin-like growth factor-1 receptor signaling pathway and causing activation of the Protein Kinase B/mechanistic target of rapamycin (AKT/mTOR) pathway (22). Luey et al. found that the type I IGF receptor is expressed widely in BC cells and mediates the effects of IGFs on cell proliferation and migration. IGF-1 activates both the PI3K/Akt and Grb2/Ras/MAP-kinase pathways to increasing the invasive capacity of BC cells (23). The abnormal activation of the classical PI3K/AKT/mTOR signaling pathway leads to a poor prognosis due to increased tumor cell proliferation, metastasis, and drug resistance. Based on these data, targeting the PI3K/AKT/mTOR signaling pathway may be a potential therapeutic strategy in the treatment of BC (24).

The regulation of key transcription factors and inflammatory mediators in the glycolytic phenotype of BC remains an area of intense investigation (25). Hypoxia is a common phenomenon within the tumor environment. The cellular hypoxic response is mediated by hypoxia-inducible factor-1 (HIF-1) which is a crucial regulatory factor of glycolysis in cancer cells. HIF-1 is an oxygen-sensing transcription factor that regulates the consumption of glucose through oxidation or glycolysis (26). Using short interfering RNA (siRNA) to silence the expression of HIF-1, Chen et al. showed that HIF-1 KO significantly inhibited the extracellular acidification rate (ECAR), glucose consumption rate, and production of lactic acid in BC cells. HIF-1 silencing has also been shown to reduce the expression of metabolic enzymes and transporters in tumor cells and reversing resistance to apoptosis in BC cells following chemotherapy treatment (27).

Hypoxia-inducible factor 1α-induced glycolysis is essential for the activation of inflammatory macrophages. For example, the high infiltration of M2 tumor-associated macrophages is an extremely important feature of inflammatory BC (28). HIF-1 regulates metabolism during hypoxia and its transcriptional activity is also induced by T cell activation in response to hypoxia (29). These changes promote metabolic reprogramming and lead to the upregulation of genes encoding glycolytic promoting enzymes such as pyruvate kinase (PKM1), hexokinase 2 (HK2), and GLUT1 (30–32). Upregulated expression of these genes can also produce a false hypoxic state in tumors to promote angiogenesis, migration, and metastasis (33) that are closely related to the occurrence and development of BC tumors.

**Figure 1:**
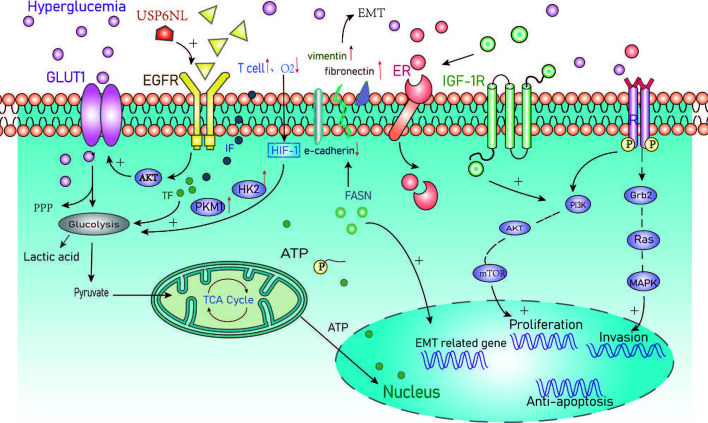
Summary of the cellular metabolic effects of hyperglycemia in cancer. GLUT1, Glucose transporter 1; PPP, Pentose phosphate pathway; EGFR, Epidermal growth factor receptor; IF, Inflammatory factors; TF, Transcription factors; HIF, Hypoxia-inducible factor-1; HK2, Hexokinase2; PKM1, Pyruvate kinase M1; EMT, Epithelial-mesenchymal transition; FASN/FAS, Fatty acid synthase; ER, Estrogen receptor; IGF, Insulin growth factor; PI3K, Intracellular phosphatidylinositol kinase; AKT, Protein kinase B; mTOR, Mammalian target of rapamycin; Grb2, Growth factor receptor-bound protein2; MAPK, Mitogen-activated protein kinase.

Epithelial-mesenchymal transformation (EMT) is an important mechanism that promotes the migration, invasion, and metastasis of cancer cells (29, 34). Zielinska et al. showed that hyperglycemia can induce matrix-specific EMT to promote the Warburg effect by upregulating glucose uptake, lactate release, and the expression of specific glycolytic enzymes and transporters. They also found that silencing fatty acid synthase (FASN) reversed the effects of hyperglycemia on the levels of EMT markers leading to increased expression of E-cadherin and decreased the expression of vimentin and fibronectin. Upregulation of these proteins is indicative of EMT and associated with metastatic progression (34). Taken together, these studies highlight the importance of hyperglycemia in the development and progression of BC (**Figure 1**).

### Resistance to Apoptosis

P53 inhibits cellular transformation and activates tumor cell responses to chemotherapy drugs. Homeodomain-interaction protein kinase 2 (HIPK2) is a nuclear serine/threonine kinase that mediates p53-dependent apoptotic pathways in tumor cells (8). Hyperglycemic environments can trigger the degradation of the HIPK2 protein and upregulate the expression of mutant p53 to inhibit p53-induced apoptosis (35). Overexpression of mutant p53 is positively correlated with high expression of the P-glycoprotein which is a known protein biomarker of chemotherapeutic resistance. Upregulation of P-glycoprotein means can facilitate drug resistance and promote tumor progression, however, this mechanism has not been frequently reported in BC.

**Figure 2:**
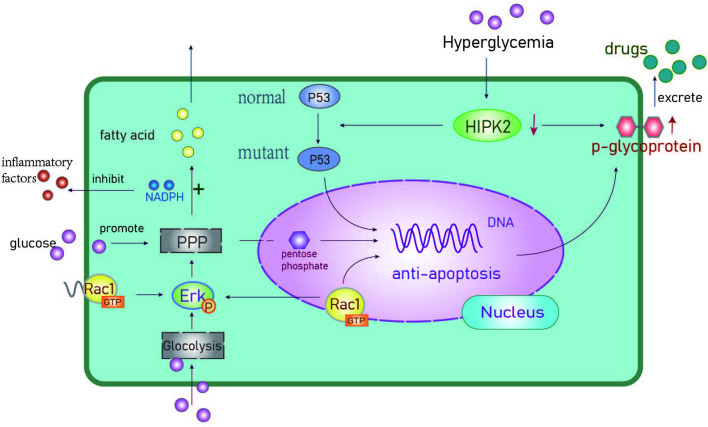
The anti-apoptotic mechanisms of hyperglycemia. Activation of anti-apoptotic genes promotes chemotherapeutic drug resistance. PPP, Pentose phosphate pathway; HIPK2, Homologous domain interacting protein kinase1; Erk, extracellular regulated protein kinase; P53, a tumor suppressor gene.

Rac1 is a small GTP binding protein. Li et al. found it is overexpressed and associated with multidrug resistance to neoadjuvant chemotherapy (NAC) (36). Rac1 activates aldosterone A and ERK signals and upregulates glycolysis, particularly the pentose phosphate pathway (PPP). This leads to an increase in nucleotide metabolism that can protect BC cells from DNA damage caused by chemotherapy (37). The PPP metabolite pentose phosphate is critical for nucleic acid biosynthesis and NADPH is essential for fatty acid synthesis as well as the mitigation of cellular oxidative stress (38) (**Figure 2**). The overexpression of Rac1 and the abnormal activation of the PPP pathway in BC patients with hyperglycemia can result in resistance to DNA damage as well as reduced oxidative stress. This can lead to therapeutic resistance that contributes to poor outcomes in BC patients.

### Resistance to Chemotherapy

Adriamycin (ADR) resistant BC cells have increased glucose metabolism (39). It has been reported that ADR containing chemotherapy plans can effectively induce insulin resistance in cells (40). The fibroblast growth factor (FGFs)-FGFR signaling pathway is involved in many biological processes such as embryonic development, wound healing, and angiogenesis. Xu et al. postulated that high expression of FGFR4 is a key regulator in ADR resistant cells that is associated with poor survival (41). Activation of FGFR4 signaling leads to phosphorylation of FGF receptor substrate 2 (FRS2) and further activation of downstream MAPK/ERK signaling. Pharmacological inhibition of the FGFR4-FRS2-ERK signaling pathway has been shown to decrease chemoresistance and the glycolytic phenotypes of ADR-resistant cells (42). MQA et al. hypothesized that glucose metabolism has a major impact on the expression of insulin-like growth factor binding protein2 (IGFBP-2) which is an essential regulator of the IGF signaling axis. The continuous secretion of IGFBP-2 promotes chemotherapy resistance in BC and can be abrogated by silencing of IGFBP-2 expression. This results in reversing chemotherapy resistance induced by high glucose levels and resensitizes BC cells to ADR (5). The combination of 3-BRPY (a glycolysis inhibitor) and ADR has been shown to reduce total ATP and lactic acid levels (43) yet these specific mechanisms remain to be fully understood.

Paclitaxel is a commonly used chemotherapy drug in the treatment of TNBC to which patients commonly develop resistance (44). Studies have reported the relationship between lactate dehydrogenase A (LDHA) and paclitaxel resistance. The downregulation of LDHA combined with paclitaxel with oxamate (an analogue of pyruvate) has been shown to result in a two-fold increase in the sensitivity to paclitaxel suggesting that targeted glycolytic enzymes may resensitize drug-resistant cells to paclitaxel. These data indicate that LDHA is a potential therapeutic target for overcoming paclitaxel resistance and in BC (45). Moreover, the most common mechanism of paclitaxel resistance is through drug efflux from the ATP binding cassette transporter. The energy required for drug efflux mainly comes from the glycolytic pathway and the P-glycoprotein is an important factor that mediates drug outflow causing drug resistance (46).

Cisplatin is a common chemotherapy drug to which BC patients often develop resistance mediated by the altered expression of glycolytic enzymes and glucose transporters (47). Considering the role of GLUT1 as an important glucose transporter (17, 18), GLUT1 may be involved in cisplatin resistance in BC cells under hyperglycemia. He et al. found that the overexpression of the oncogene TRIM59 in non-small cell lung cancer cells was related to cisplatin resistance and was mediated by the glycolysis-related gene HK2. Based on these data, we hypothesize that TRIM59 is involved in the metabolic reprogramming of cisplatin-resistant cancer cells by regulating the expression of HK2 (48), however, this has not yet been reported in the field of BC.

### Resistance to Endocrine Therapy and Targeted Drugs

Tamoxifen is the most commonly used endocrine therapy in BC and patients often develop drug resistance due to enhanced glycolysis in ER-positive BC. He et al. demonstrated that activation of the EGFR signaling pathway and its downstream glycolytic genes play an important role in tamoxifen-resistant BC cells (49). Tamoxifen acts by inhibiting the mitochondrial respiratory complex I to reduce ATP levels and activate AMPK. These alterations induce apoptosis by suppressing mTOR (50). Hyperglycemia can activate the AKT/mTOR/AMPK signaling pathway which is involved in tamoxifen resistance (51). Also, Huang et al. recently reported that tamoxifen inhibits the proliferation of gallbladder cancer cells by impairing glucose metabolism (52). It is known that the occurrence of gall bladder cancer may be related to estrogen receptors suggesting a potential link with the effects of tamoxifen. This deserves in the field of breast cancer deeply.

**Figure 3:**
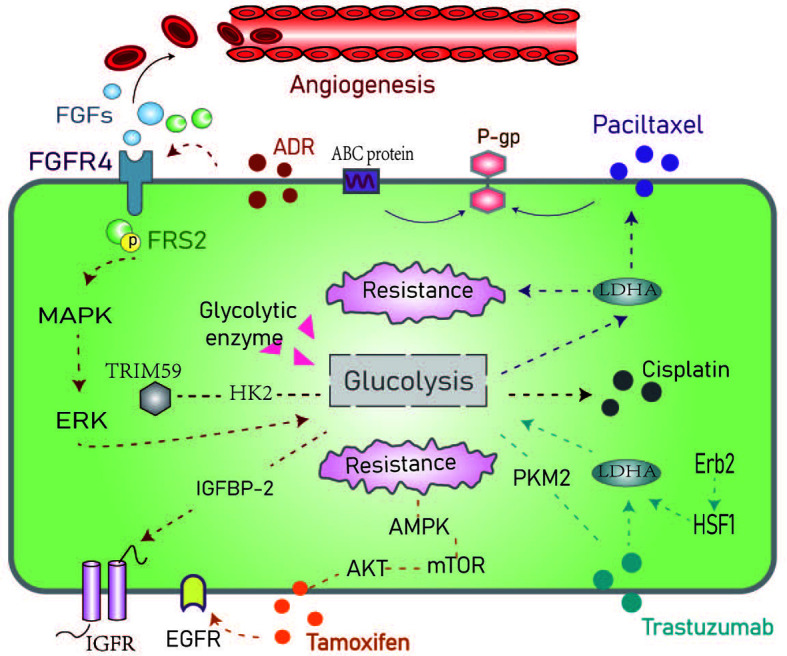
Schematic representation of the resistance mechanisms of common chemotherapeutic drugs in the hyperglycemic environment. The dotted line represents the route in the text. ADR, Adriamycin; FGF/FGFR, Fibroblast growth factor and its receptor; FRS, Fibroblast growth factor receptor substrate; IGFBP-1, Insulin-like growth factor-binding protein-1; ABC protein, ATP binding cassette transporter; P-glycoprotein; AMPK, AMP-activated protein kinase; PKM2, Pyruvate kinase M2; HSF1, Heat shock factor1; LDHA, Lactate dehydrogenase.

Trastuzumab (Herceptin) is an antibody targeted against HER2. Aerobic glycolysis can be inhibited by the ErbB2 (HER2)-heat shock factor1 (HSF1)-LDHA pathway. The sensitivity to trastuzumab has been associated with LDHA activity (53). PKM2 is another key glycolytic enzyme that has been implicated in response to trastuzumab in BC (54). PKM2 is a rate-limiting enzyme in glycolysis that has been proposed as an early marker of the treatment response to trastuzumab in BC patients with metastasis (55). The PI3K/AKT signaling pathway has multiple control points in the glucose metabolism pathway including glucose transporters and enzymes that regulate glycolysis. Studies have proposed that trastuzumab combined with PI3K/AKT inhibitors may be used to improve responses to treatment in cancer (56). In particular, one study showed differences in glucose metabolism between ER+/HER2−/+ subtypes of BC cell lines exposed to palbociclib. In ER+/HER2-palbociclib sensitive cells, aerobic glycolysis and glucose catabolism were enhanced in ER+/HER2+ Palbociclib resistance (57) (**Figure 3**). However, the mechanism through which targeted drugs promote aerobic glycolysis in BC remains to be fully determined.

## The Effects of Hyperglycemia in the Tumor Microenvironment (TME)

BC cells undergo metabolic reprogramming that usually includes enhanced glycolysis and increased activity of the tricarboxylic acid cycle (58). Scholars put forward the blood glucose changes three pathways in TME: VEGF and its receptors, cell to cell, and cell to extracellular matrix (ECM) adhesion proteins. What cause BC cells (MDA-MB-231) to metastatic mutations to bone and brain (59). It can be seen that the blood glucose load in the microenvironment is very important in tumor growth. We talked about the changes caused by hyperglycemia in the microenvironment, such as the increase in PH, the high concentration of lactic acid, the production of inflammatory factors, the imbalance of ROS, and the impact on immune cells.

### The Abnormal Microenvironment

The TME is mainly composed of tumor cells, surrounding immune and inflammatory cells, fibroblasts, interstitial tissue, capillaries, various cytokines, and chemokines. The TME is characterized by high levels of H2O2 and glutathione (GSH), low pH, and hypoxia that have important implications for responses to treatment (60).

The TME plays an important role in the metabolic adaptation and survival of tumor cells. The inflammatory microenvironment can directly induce aerobic glycolysis (61). Studies have found that hyperglycemia and insulin can induce mesenchymal phenotypes through the generation of reactive oxygen species (ROS) (62). Under hyperglycemic or pathological microenvironments, imbalances between the production and removal of ROS and the production of chronic inflammatory markers (such as IL6, TNF-α, and COX-2) under hyperglycemic conditions can induce anti-apoptotic activities and EMT in cells (63).

The Warburg effect causes tumor cells to continuously export and accumulate lactic acid in the TME (5, 64). Chen and colleagues reported that high levels of lactic acid are related to the incidence of distant metastasis. LIN28B promotes the secretion of lactate and enhances the stem cell properties of cancer cells. Overexpression LIN28B increases the rate of extracellular acidification, glucose uptake, and lactic acid secretion both *in vivo* and *in vitro* (65). The impact of the acidic TME on cancer stem cells (CSCs) is thought to result in tumor relapse, therapeutic resistance, and metastasis (66). Moreover, lactic acid is also a key factor involved in angiogenesis and immune evasion. Lactic acid leads to extracellular pH acidification within the microenvironment that is also related to clinical prognosis (67). The production of lactic acid depends on the levels of key enzymes in the glycolytic pathway. LDH also plays an important role in regulating the nutritional exchange between tumor cells and stroma cells. Studies have found LDHA targeting tumor cells and LDHB targeting stromal cells influence tumor proliferation. These data suggest that it may be beneficial to block lactate exchange between tumor and stroma as a potential therapeutic strategy (68).

**Figure 4:**
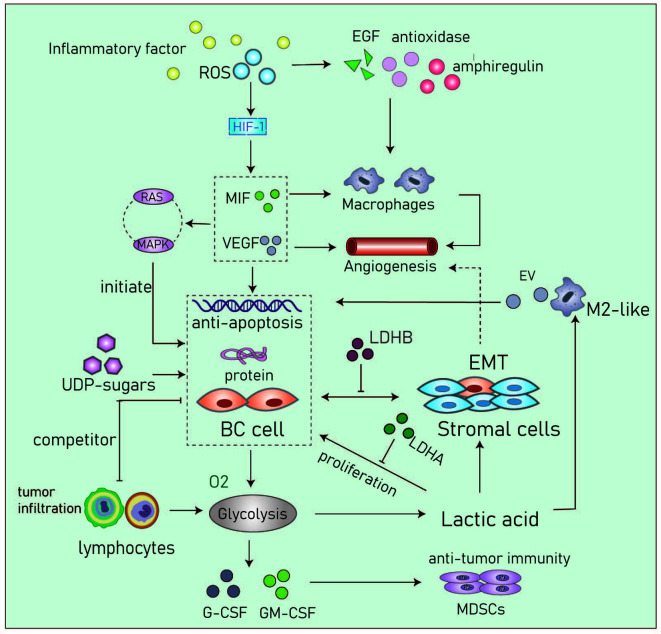
Cancer cells in the acidic microenvironment can escape the immune system and eventually lead to tumor progression. Inflammatory factor, IL6, TNF-α, COX-2 and so on; RDS, Reactive oxygen species; MIF, Migration inhibitory factor; VEGF, Vascular endothelial growth factor; UDP-sugar, Uridine diphosphate glucose; G-CSF, Granulocyte colony stimulating factor; GM-CSF, Granulocyte macrophage colony stimulating factor; MDSCs, Myeloid-derived suppressor cells; EV, Extracellular vesicle, transmit myeloid-specific lncRNA and HIF-1α-stabilizing lncRNA.

Uridine diphosphate glucose (UDP)-sugars are generated as intermediate products of glucose metabolism. The levels of these sugars are significantly increased in BC and have been shown to promote the accumulation of hyaluronic acid which is a known promoter of the disease. The metabolism of hyaluronic acid within the TME a key factor that drives invasive growth and metastasis (69, 70). In ductal and lobular BC, the levels of UDP-sugars are significantly increased. These observations suggest that blocking the excessive supply of UDP-sugars and reducing the content of hyaluronic acid may be potential therapeutic strategies in BC patients with glucose metabolism disorders (71, 72) (**Figure 4**).

Cancer cells within the TME have higher levels of reactive oxygen species (ROS) compared to normal cells. In basal-like and BRCA1-associated BC, it has been shown that ROS levels are associated with the expression and activity of the transcription factor aryl hydrocarbon receptor (AHR). These changes promote the transcription of antioxidant enzymes, epidermal growth factor receptor (EGFR) ligands, and the bidirectional regulatory factor AREG. AHR can attract monocytes into the TME and activate macrophages to promote angiogenesis (73, 74). Parekh et al. captured multinucleated cells in chemotherapy-resistance triple-negative BC cells. They showed that these cells are non-proliferate but can significantly regulate the TME by elevating levels of ROS and by stabilizing HIF-1. These processes contribute to increased levels of vascular endothelial growth factor (VEGF) and macrophage migration inhibitory factor (MIF) and can induce chemotherapeutic resistance by upregulating anti-apoptotic proteins through the RAS/MAPK pathway (75).

### Changes in Immune Cells Within the TME

The highly acidic TME formed by glycolysis may affect the infiltration of immune cells eventually leading to immune escape and cancer progression (76). The immune infiltrates of the TME consist of lymphocytes and bone marrow cells. In this section, we briefly review the changes in immune cells in the hyperglycemia TME.

Macrophages are the most abundant type of immune cell found in the TME. M2 macrophages are closely related to the occurrence and development of tumors. Lactic acid can promote macrophages towards an M2-like phenotype that is associated with adverse clinical characteristics such as large tumor volumes, higher histological grades, ER negativity, higher recurrence rates, and lower rates of survival (76, 77). It has been demonstrated that tumor-associated macrophages (TAMs) enhance aerobic glycolysis and apoptotic resistance in BC cells *via* the transmission of extracellular vesicles (EV) that contain a myeloid-specific HIF-1α-stabilizing long noncoding RNA (HISLA). Lactic acid released by glycolytic tumor cells upregulates macrophage HISLA and forms a feed-forward loop between TAMs and tumor cells in the TME (27).

Tumor-infiltrating lymphocytes (TIL) comprise B and T cells. Cytotoxic CD8+T lymphocytes (CTL) are the most abundant TILs in the TME of BC but helper CD4+T cells and NK cells are also present (78). Tumor cells compete with these cells for glucose. Abundant lactate production by tumor cells has been shown to inhibit MCT1-mediated lactate export by TILs leading to decreased cell proliferation, cytokine production, and/or cytolysis. This phenomenon will inhibit the glycolysis of T cells and inhibit their tumor-killing function along with functional damage to NK cells (79).

Myeloid-derived suppressor cells (MDSCs) inhibit anti-tumor immunity. In TNBC mouse models, studies have found glycolysis inhibits the expression of granulocyte colony-stimulating factor (G-CSF) and granulocyte macrophage colony-stimulating factor (GM-CSF) resulting in the decreased expression of MDSCs and promotion of tumor immunosuppression (80). Although other immune cells such as dendritic cells, mast cells and granulocytes are also present in the BC TME, studies are required to better define their metabolic interactions with tumor cells (**Figure 4**).

## Hyperglycemia Affects Response to Therapy in BC Patients

### Anti-Sugar Drugs in BC Patients

In BC patients, hyperglycemia during chemotherapy will increase the resistance to treatment (81) and so reducing blood glucose levels is particularly important. Drugs that alter glucose metabolism can be beneficial in improving the effects of conventional chemotherapy drugs commonly used in cancer treatment. The choice of chemotherapy plan and cycle time should be carefully considered in hyperglycemic patients.

Metformin was developed in the late 1970s as an anti-hyperglycemia drug (82). It has now been widely tested for an anticancer agent along with other hyperglycemic drugs (3). Metformin can alter cancer metabolism and mitochondrial function. It can also regulate key signaling pathways such as the Ras/Raf MEK/ERK PI3K/Akt and mTOR pathways to increase cell death and inhibit many cellular processes including proliferation, migration, EMT, invasion, and metastasis (83). Metformin also changes signal transmission of the Warburg effect during tumor development and can inhibit glucose uptake by cancer cells (84). It reduces circulating hormone levels, particularly estrogens, that are associated with the development of postmenopausal BC (85).

Additionally, studies have shown that the thiazolidinediones are insulin sensitizers that can also inhibit the growth of BC cells. Pioglitazone acts as an agonist of the tumor suppressor PPAR. When PPAR is activated, the levels of free fatty acid (FFA) and eicosanoid are reduced, and VEGF-induced angiogenesis is inhibited. Also, the proliferation and migration of cancer cells are inhibited through the JAK2/STAT3 pathway (86).

Insulin and insulin analogs such as sulfonylureas and glitinides have powerful hypoglycemic effects. Studies have reported that insulin downregulates IGF-BPs and sex hormone-binding proteins (SHBGs) leading to IGF and hormone-dependent BC (21). Type 2 diabetes and insulin therapy may be independently associated with a poorer prognosis in BC. Premenopausal women with diabetes tend to develop breast tumors that do not express hormone receptors making their treatment extremely challenging (87, 88).

The antidiabetic drugs described above may also have potential roles as anti-cancer drugs. A total of 46 studies of metformin in BC patients have been registered in ClinicalTrials.gov. 17 studies have completed, 14 studies are recruiting, 4 studies were terminated due to inapplicable factors such as long rest intervals, changes in treatment methods, slow accumulation of patients’ number, and data loss. And we also have listed completed in **Table 1** and recruiting trials in **Table 2**. Hope to be helpful to interested readers.

**Table 1:** Completed researches on ClinicalTrials.gov. We excluded some studies that have too few participants, or inapplicable conclusions.

Phase	Study Design	Number
II	**group1**: metformin; **group2**: metformin plus chemotherapy	NCT04143282
II	**group1**: metformin; **group2**: placebo	NCT01310231
II	**group1**: exercise training; **group2**: exercise training with metformin; group3: only metformin; group4: control	NCT01340300
II	**group**: Liposomal doxorubicin +Docetaxel+Trastuzumab+Metformin	NCT02488564
II	**group1**: Letrozole with concurrent metformin; **group2**: Letrozole with placebo	NCT01589367(**94**)
II	**group1**: placebo; group2: Metformin 500 mg/d; group3: Metformin 1,000 mg/d	NCT00909506
II	**group1**: Metformin + Myocet + Cyclophosphamide; **group2**: Myocet + Cyclophosphamide	NCT01885013
I	**group1**: Exemestane alone; **group2**: Exemestane plus metformin plus rosiglitazone	NCT00933309

**Table 2:** Recruiting researches on ClinicalTrials.gov.

Phase	Study Design	Registration no. on ClinicalTrials.gov accessed November 10, 2020
I	**metformin group**: GDC-0077+Palbociclib+Fulvestrant+Metformin; **control group**: GDC-0077 + Palbociclib + Fulvestrant	NCT03006172
II	**metformin group**: Dexamethasone and Metformin; **control group**: Dexamethasone	NCT04001725
II	**metformin group**: Toremifene and metformin; **control group**: Toremifene	NCT02506790
II	**metformin group**: 5 – Fluoruracil, doxorubicin, cyclophosphamide(FDC) ×6 cycles with metformin; **control group**: FDC ×6 cycles	NCT02506777
II	**metformin group**: Fasting-mimicking diet plus metformin plus chemotherapy **control group**: Chemotherapy plus Fasting-Mimicking Diet (FMD)	NCT04248998
II	**metformin group**: Taxotere, Carboplatin, Herceptin + Pertuzumab (TCH+P) plus metformin; **control group**: Taxotere, Carboplatin, Herceptin + Pertuzumab	NCT03238495
II	**metformin group**: receive AC-T neoadjuvant chemotherapy in addition to oral metformin HCl (850 mg tablets, twice per day, for 6 months) **control group**: receive AC-T neoadjuvant chemotherapy alone	NCT04170465
II	**metformin group**: 4 cycles (Doxorubicin+Cyclophosphamide) followed by 12 cycles Paclitaxel+ Metformin (1,000 mg twice daily) followed by surgery. **control group**: 4 cycles (Doxorubicin+Cyclophosphamide) followed by 12 cycles Paclitaxel followed by surgery.	NCT04559308
II、III	**metformin group**: metformin 850 mg once daily increased within 3 weeks to a maximum dose of 2,550 mg on three divided daily doses. Neoadjuvant cytotoxic chemotherapy as per MDT (multi-disciplinary team) decision. Patients scheduled for AC-T (adriamycin, Cyclophosphamide, paclitaxel) or AC (adriamycin, cyclophosphamide) will be eligible to randomization. **control group**: placebo plus the some neoadjuvant	NCT04387630
III	**metformin group**: receive metformin hydrochloride PO QD or BID for 24 months. Patients will continue metformin 850 mg PO BID for months 13–24; **control group**: receive placebo PO QD or BID for 12 months. Patients may crossover to Arm I for months 13–24.	NCT01905046
I	**metformin group**: metformin **control group**: Atorvastatin	NCT01980823
II	**metformin group:** Metformin Hydrochloride **control group:** Doxycycline	NCT02874430
I	**I-SPY trial**: Multi-group study, one group of metformin intervention	NCT01042379
II	**METALLICA**: Normal fasting glycemia group: Alpelisib plus metformin and fulvestrant Abnormal fasting glycemia group: Alpelisib plus metformin and fulvestrant	NCT04300790

Combining the above table, we can see that the role of metformin on its anti-tumor effects are researched continuously. In addition, there are reported retrospective studies in the past have shown that the use of metformin combined with neoadjuvant chemotherapy can enable BC patients to obtain a higher pCR rate (89). The METTEN study shows that the addition of metformin plus trastuzumab to neoadjuvant chemotherapy can effectively increase the pCR rate of early her2-positive BC patients (90). The METEOR study also provides evidence of the neoadjuvant metformin plus letrozole for anti-tumor effects in non-diabetic postmenopausal ER-positive patients (91).

Although metformin has shown good antitumor activity in BC, there remain many challenges concerning how best to further optimize treatment. The efficacy of metformin as an anticancer drug depends largely on the glucose concentration in the TME and so treating diabetes in cancer patients may be necessary. There are relatively few studies concerning glitazones and other antidiabetic drugs. The heterogeneity and differences in diseases mean that it is necessary to adopt personalized treatments and using precision medicine approaches to tailor treatments at the individual patient level.

Patients with hyperglycemia are resistant to chemotherapy drugs. Metformin resistance is no exception (92). Scherbakov et al. demonstrated for the first time that structural activation of Akt/Snail1/E-Cadherin signaling leads to cross-resistance of BC cells to metformin and tamoxifen (93).

### Glycolysis Inhibitors in BC Patients

Reports have shown that glycolysis inhibitors such as 2-deoxy-d-glucose (2DG) combined with metformin can also significantly reduced the survival of BC cells (94). It has been reported in the literature that the combined treatments using glycolysis inhibitors and anti-glycemic drugs are feasible in humans, however, these approaches have not yet translated to clinical evaluation.

### Targeted Drugs in BC Patients

PI3K/AKT/mTOR and CDK4/6 inhibitors are emerging drugs for the treatment of ER-positive and human epidermal growth factor receptor-2 (HER2) negative metastatic BC (95). The PI3K/AKT/mTOR pathway is an important oncogenic signaling pathway in BC that can be activated by hyperglycemia to promote the proliferation of malignant BC epithelial cells (56, 95). Everolimus is an mTOR inhibitor that can target the rapamycin pathway. However, because of its toxic effect and its efficacy it has limited applications in the clinic (96). Gerke et al. have found that everolimus combined with metformin had a combined anti-cancer effect and a common inhibitory effect on glucose metabolism, tumor cell growth, and colony formation (97). However, hyperglycemia is also the most common adverse reaction of everolimus (98). Metformin may be used to prevent and/or treat everolimus-induced hyperglycemia and may enhance its anticancer effects yet there are relatively few clinical research studies in this area.

CDK4/6 inhibitors have rapidly translated from preclinical studies to clinical evaluation (99). Palbociclib can downregulate glucose uptake by GLUT-1 through the RB/E2F/C-MYC signaling pathway to reprogram glucose metabolism. It also inhibits the expression of HIF-1, a key regulator of tumor progression (100). *In vitro* experiments have shown that ER+/HER2- BC cells are sensitive to palbociclib under conditions of enhanced aerobic glycolysis whilst ER+/HER2+ cells show enhanced glycolytic catabolism with the development of palbociclib resistance. These metabolic phenotypes may have potential prognostic value (101).

## Conclusions

Abnormal glucose metabolism is an important clinical problem in many types of BC. Recent studies have shown that somatic and BC cells from patients with hyperglycemia or metabolic abnormalities have elevated acidity in the TME accompanied by increased ROS and other changes in energy homeostasis. Hypoxia within the TME leads to the abnormal activation of cancer cell behaviors including enhanced proliferation, invasion, and metastasis. The reprogramming of metabolic changes in the glycolysis pathway increases the supply of substrates to cancer cells. However, tumor cells need to adapt to new conditions within the TME which can affect the survival and prognosis of BC patients.

The treatment of BCs with abnormal metabolism remains a major clinical challenge. For example, metformin can be used as an anticancer drug and can also control hyperglycemia or diabetes in patients. However, the efficacy of metformin depends on the glucose concentration within the TME and the sensitivity of patients to metformin. Other anti-sugar drugs also have anti-cancer effects but have not been studied in detail. Consequently, individualized treatment plans are required to optimize BC treatments in patients with hyperglycemia.

## Author Contributions

JQ and QZ contributed equally to this paper. JQ wrote the manuscript and composed the figures for this article. QZ reviewed the literature and contributed to the writing of the article. XM contributed to the editing and composition of the final version. All authors contributed to the article and approved the submitted version.

## Funding

This work was supported by a special program from the Chinese National Natural Science Funds (81973861 to XM), the Key Construction Project Co-sponsored by Province and Ministry (WKJ-ZJ-2116 to XM), the Key Project for Medical and Health in Zhejiang Province (2016ZDA004 to XM), Zhejiang Medicine and Health Technology Plan Project(WKJ-ZJ-1803-01-XM), the Medical and Health Projects in Zhejiang Province (2020KY495 to QZ) and Zhejiang Traditional Chinese Medicine Project(2020 ZQ 009-QZ).

## Conflict of Interest

The authors declare that the research was conducted in the absence of any commercial or financial relationships that could be construed as a potential conflict of interest.
